# Rapid resolution of refractory cutaneous lichen planus with abrocitinib

**DOI:** 10.1016/j.jdcr.2026.01.002

**Published:** 2026-01-10

**Authors:** Andy Ho, Rebecca Goldberg, Carsten R. Hamann

**Affiliations:** a Mayo Clinic Alix School of Medicine, Phoenix, Arizona; b HonorHealth Dermatology Residency Program, Scottsdale, Arizona; c Department of Dermatology, Dartmouth-Hitchcock Medical Center, Lebanon, New Hampshire

**Keywords:** abrocitinib, JAK inhibitor, lichen planus

## Introduction

Lichen planus (LP) is an inflammatory skin condition characterized by purple to violaceous, polygonal, shiny, flat-topped papules and plaques often with a superimposed, reticulated white scale, known as Wickham’s striae. These lesions are typically extremely pruritic with possible involvement of the skin, oral mucosa, genitalia, and/or hair and nails. First-line treatment for cutaneous lichen planus includes topical corticosteroids with refractory cases treated with systemic glucocorticoids, phototherapy, or oral retinoids.^1^ A recent systematic review studying the use of Janus kinase (JAK) inhibitors in treating LP, including tofacitinib, baricitinib, ruxolitinib, and upadacitinib, found a mixed response with 73.3% achieving partial or complete resolution and 26.7% achieving no resolution.^2^ However, there are limited data in the use of abrocitinib, another JAK inhibitor, in the treatment of cutaneous LP. This report presents a case of a patient with treatment-refractory cutaneous LP with rapid near resolution of lesions after a 2-week treatment with abrocitinib.

## Case report

A 76-year-old female presented with a pruritic rash and sores over her arms and legs present for 2 months. Physical examination showed purple to violaceous, polygonal, shiny, flat-topped firm papules and plaques with evidence of Wickham’s striae over the bilateral upper and lower extremities. No mucosal involvement was seen on exam. Topical triamcinolone 0.1% cream and hydrocortisone 1% cream were previously ineffective in treating the lesions. A 2-week oral prednisone 40 mg taper was started with recommendations to continue topical triamcinolone and hydrocortisone.

The patient returned 3 months later with worsening lesions spreading to her upper back. Infectious panel including coccidiomycosis, hepatitis, and tuberculosis were negative. A lichenoid drug eruption was considered as the patient was taking chlorthalidone and metoprolol; therefore, biopsy was obtained which was consistent with lichen planus. Another 3-week prednisone 60 mg taper and topical clobetasol 0.05% ointment were started, but the patient continued to report minimal relief.

**Fig 1:**
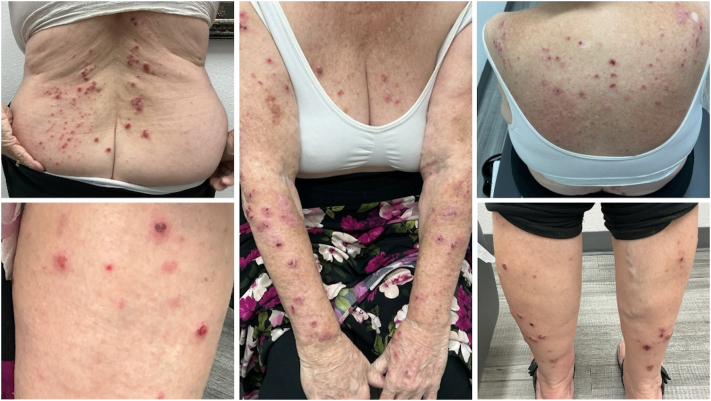
*Purple* to violaceous papules with overlying excoriations and erosions involving the chest, back, and bilateral upper and lower extremities.

**Fig 2:**
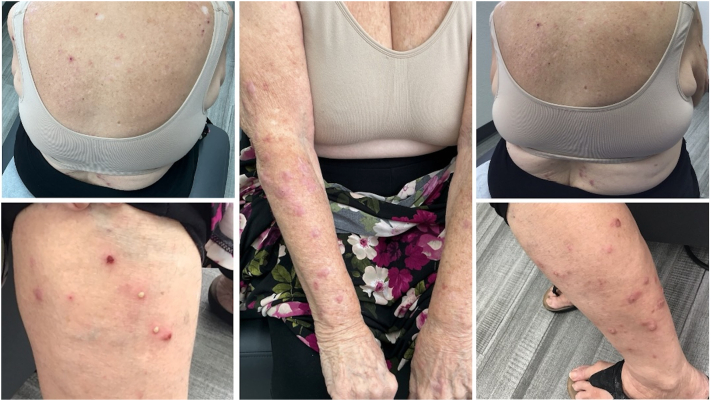
Improving papules and patches 2 weeks after initiation of abrocitinib.

**Fig 3:**
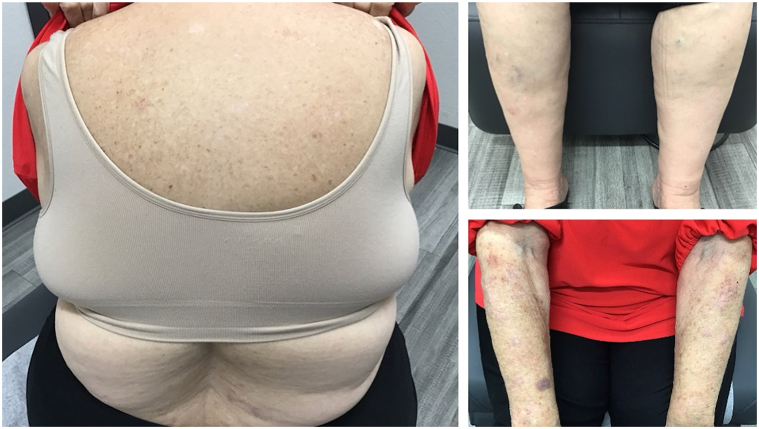
Continued improvement and resolution of papules and patches 2 months after treatment with abrocitinib.

Nine months after initial presentation, the patient reported persistent pruritic papules and plaques, thus she was initiated on topical betamethasone 0.05% cream, metronidazole 500 mg by mouth twice daily, doxepin 3 mg by mouth at bedtime PRN, and a 6-week 60 mg prednisone taper. Although the patient experienced significant relief after an extended oral steroid taper, her lesions continued to recur. The patient continued to have worsening papules and plaques 15 months after initial presentation despite repeated oral prednisone tapers, high-potency topical corticosteroid therapies, oral metronidazole, and discontinuation of chlorthalidone (Fig 1). Her treatment course was also complicated by COVID pneumonia. After discussion of risks and benefits with the patient, treatment with abrocitinib 100 mg daily was initiated. After 2 weeks of treatment with abrocitinib, the patient reported significant improvement in her pruritus and the appearance of her lesions (Fig 2). Two months after the introduction of abrocitinib, the patient had complete resolution of her lichen planus with no pruritus and reported continued improvement in the appearance of her lesions (Fig 3). She denied any adverse effects from abrocitinib and noted improvement in her overall quality of life, self-esteem, and body image. Topical triamcinolone was recommended for any remaining open sores. Four months after initiation of therapy, the patient continues to use abrocitinib with no new lesions and no reported adverse effects.

## Discussion

Although the exact mechanism is not understood, the pathogenesis of LP is believed to be driven by a cell-mediated immune response from cytotoxic CD8+ T-cells. Increased serum levels of several cytokines and chemokines have also been found in patients with LP, including interferon-gamma, which activates the Janus kinase (JAK)/signal transducers and activators of transcription pathway.^1^ Cytokines further activated by the JAK-signal transducers and activators of transcription pathway have been theorized as a contributor to the pathogenesis of LP.^3^ Thus, JAK inhibitors blocking this pathway present as a possible therapeutic option.^1^

The findings in this case support a similar previously reported case involving a patient with disseminated cutaneous LP, in which abrocitinib led to complete resolution of lesions within a month after failure of multiple therapies. Abrocitinib has also been reported to treat refractory vulvar, oral, and nail LP with excellent clinical response, subjective patient improvement, and minimal adverse effects.^4^, ^5^, ^6^, ^7^ Compared to other nonselective JAK inhibitors, abrocitinib is a selective JAK1 inhibitor, potentially minimizing the risk of anemia and neutropenia.^6^^,^^8^ JAK1 has been found to be overexpressed in LP which, in addition to its effects on immune modulation, could explain the therapeutic benefits of abrocitinib.^9^

Several case reports and series have been published regarding the effective use of JAK inhibitors in the treatment of LP, and Motamed-Sanye et al found them to be efficacious in treating LP variants including oral LP, erosive LP, and lichen planopilaris.^10^ However, studies have been limited to case reports and series with even more limited reports on abrocitinib. Additional randomized controlled trials and longitudinal studies are needed to further evaluate the efficacy and safety of abrocitinib in the treatment of cutaneous LP. This case suggests that abrocitinib can be a safe and effective option for cutaneous LP refractory to first-line treatment options.

## Conflicts of interest

Dr Hamann has worked as a consultant for and received honoraria from Pfizer. Dr Goldberg and Author Ho have no conflicts of interest to declare.

## References

[1] Boch K., Langan E., Kridin K., Zillikens D., Ludwig R., Bieber K. (2021). Lichen planus. Front Med.

[2] Abduelmula A., Bagit A., Mufti A., Yeung K.Y., Yeung J. (2023). The use of Janus kinase inhibitors for Lichen planus: an evidence-based review. J Cutan Med Surg.

[3] Cinats A., Heck E., Robertson L. (2018). Janus kinase inhibitors: a review of their emerging applications in dermatology. Skin Ther Lett.

[4] Barbosa F., Siems L., Luz D., Duarte A. (2025). Targeting JAK-1 in disseminated cutaneous Lichen planus. JEADV Clin Pract.

[5] DeBiasio C., Kirshen C. (2025). Recalcitrant vulvar Lichen planus cleared on abrocitinib: a case report. SAGE Open Med Case Rep.

[6] Solimani F., Mesas-Fernández A., Dilling A. (2023). The Janus kinase 1 inhibitor abrocitinib for the treatment of oral Lichen planus. J Eur Acad Dermatol Venereol.

[7] He J., Yang Y. (2024). Janus kinase 1 inhibitor abrocitinib for isolated nail lichen planus: a case report and literature review. J Dermatol Treat.

[8] Taylor P., Choy E., Baraliakos X. (2024). Differential properties of Janus kinase inhibitors in the treatment of immune-mediated inflammatory diseases. Rheumatology.

[9] Alves de Medeiros A., Speeckaert R., Desmet E., Van Gele M., De Schepper S., Lambert J. (2016). JAK3 as an emerging target for topical treatment of inflammatory skin diseases. PLoS One.

[10] Motamed-Sanaye A., Khazaee Y.F., Shokrgozar M., Alishahi M., Ahramiyanpour N., Amani M. (2022). JAK inhibitors in lichen planus: a review of pathogenesis and treatments. J Dermatol Treat.

